# *Myristica fragrans* Houtt. methanol extract as a promising treatment for *Cryptosporidium parvum* infection in experimentally immunosuppressed and immunocompetent mice

**DOI:** 10.14202/vetworld.2024.2062-2071

**Published:** 2024-09-15

**Authors:** Eman E. El Shanawany, Faten Abouelmagd, Noha Madbouly Taha, Rabab S. Zalat, Enas H. Abdelrahman, Eman H. Abdel-Rahman

**Affiliations:** 1 Department of Parasitology and Animal Diseases, National Research Centre, Dokki-Giza, Egypt; 2 Department of Medical Parasitology, Faculty of Medicine, Sohag University, Sohag, 82524, Egypt; 3 Department of Parasitology, Kasr Al-Ainy School of Medicine, Cairo University, Egypt; 4 Department of Parasitology, Theodor Bilharz Research Institute, Egypt; 5 Department of Pharmacognosy, Faculty of Pharmacy, Cairo University, Kasr El-Aini Street, Cairo, Egypt

**Keywords:** *Cryptosporidium parvum*, gas chromatography-mass spectrometry, immunosuppressed mice and immunocompetent mice, *Myristica fragrans* Houtt, treatment

## Abstract

**Background and Aim:**

Cryptosporidiosis is a major waterborne disease affecting ruminants and humans worldwide. It causes diarrhea and neonatal mortality in buffalo calves, and watery diarrhea and mortality in children and immunodeficient patients. This study aimed to investigate the efficacy of *Myristica fragrans* methanolic extract in treatment of *C. parvum* infection in comparison with nitazoxanide (NZX) (a Food and Drug Administration-approved drug control) in immunosuppressed and immunocompetent mice.

**Materials and Methods:**

One hundred laboratory-bred male Swiss albino mice were equally divided into immunocompetent and immunosuppressed groups. Each group was further divided into five subgroups: (1) non-infected and non-treated control, (2) infected and non-treated control (infected with *Cryptosporidium* parvum oocysts 3 × 10^3^), (3) NZX-treated (100 mg/kg, 200 μL/mouse), (4) *M. fragrans* Houtt. methanol extract-treated (500 mg/kg), and (5) combination-treated (NZX + *M. fragrans* extract). Number of oocysts/g of feces, serum immunoglobulin (Ig) G level, and interferon (IFN)-γ, and interleukin (IL)-4 levels were used to evaluate the therapeutic effect.

**Results:**

*C. parvum* oocyst shedding in stool samples was significantly decreased in all treatment groups, with 79.7%, 81.2 %, and 85.5 % reduction in immunocompetent mice treated with NZX, *M. fragrans*, and their combination, respectively. In immunosuppressed mice, oocyst shedding was reduced by 77.7%, 80.5 %, and 83.7 % upon NZX, *M. fragrans*, and their combination treatments, respectively. The serum IgG level was lowest in mice treated with a mixture of *M. fragrans* and NZX, followed by those treated with NZX, and was highest in mice treated with *M. fragrans* alone. Regarding cytokine levels, all groups treated with *M. fragrans* had low levels of IFN-γ and IL4 on day 21 post-infection.

**Conclusion:**

Collectively, the treatment of cryptosporidiosis with *M. fragrans* extract was successful in mice, as demonstrated by the measured parameters. *M. fragrans* reduced *C. parvum* oocyst shedding and serum IgG, IFN-γ, and IL-4 levels in immunocompetent and immunosuppressed mice.

## Introduction

Cryptosporidiosis is a major waterborne disease that occurs worldwide. *Cryptosporidium parvum* is one of the most important *Cryptosporidium* species and causes serious health problems in many vertebrate hosts, including humans and ruminants. In ruminants, *C. parvum* infection results in diarrhea, lack of appetite, fever, and malabsorption. In neonatal livestock, *C. parvum* infection represents a serious health problem, causing decreased growth rates and high mortality, as well as increasing the cost of animal healthcare and veterinary services, leading to economic losses [1]. In humans, *C. parvum* infection causes watery diarrhea that affects immunocompetent and immunocompromised individuals, leading to high mortality in both children and immunocompromised patients, especially those infected with human immunodeficiency virus (HIV) [2]. Cryptosporidiosis was declared in the “World Health Organization’s Neglected Disease Initiative,” which included important infectious diseases affecting people in developing countries because of poverty, climatic factors, and the inability to access medical services. Therefore, collaborative veterinary and medical efforts should be made to control cryptosporidiosis [3]. Cell-mediated immune responses in the form of interferon (IFN)-γ production play crucial roles in the outcome of cryptosporidiosis [4]. Low CD4^+^ T-lymphocyte counts are associated with a higher prevalence of cryptosporidiosis, including persistent diarrhea, severe dehydration, malnutrition, weight loss, and even death [5]. The humoral immune response is represented by serum immunoglobulin (Ig) M level on day 10 and IgG production on day 14 post-infection (p.i.), also plays a role [6]. Cryptosporidiosis control is considered a “One Health” goal that requires harmonized efforts directed toward its prevention in humans and livestock. However, there are limited treatment options for cryptosporidiosis [7]. Only nitazoxanide (NZX) has been approved by the Food and Drug Administration (FDA) for managing cryptosporidiosis in humans. However, the cure rate of NZX is low in malnourished children and immunocompromised individuals. In ruminants, only halofuginone lactate has been approved for the treatment of cryptosporidiosis, but with limited efficacy [8]. Therefore, it is necessary to develop alternative drugs with improved safety and efficacy [9].

Herbal and alternative medicines have great potential for treating parasitic diseases, and their derivatives are useful for drug synthesis and bioactivity optimization [10–12]. Nutmeg, *Myristica fragrans* Houtt., is a large, leafy tree that originates from Moluccas Island in Indonesia and is known as “the spice island.” Nutmeg has been used to treat anxiety, dyspepsia, cramps, nausea, and diarrhea [13]. It is also used as a food preservative because of its antimicrobial activity [14]. Myristicin is the largest constituent of nutmeg and is thus known to be a beneficial constituent, although it is also responsible for some aspects of nutmeg’s toxicity [15]. It is composed of a safrole derivative with a methoxy group at carbon 4. The methoxy group bestows myristicin with a strong tranquilizing effect [16]. In addition, myristicin is metabolized in the human body to the metabolite “3-methoxy-4,5-methylenedioxyamphetamine,” which is known as a strong sedative and is associated with locomotor inhibition in mice [17, 18]. Several studies have identified the therapeutic properties of myristicin as an antioxidant, antiproliferative antimicrobial, insecticide, and larvicide [19–22]. *M. fragrans* is also known to exhibit antidiabetic [23] and lipid-lowering effects [24]. Moreover, *M. fragrans* affects the central nervous system; it is used in alternative medicine as a memory potentiator [25]. *Myristic fragrans* also exert antidepressant effects [26] and enhance learning capacity [27].

With regard to *M. fragrans* ethnopharmacology and the significance of natural sources as potential therapeutic candidates, there is an urgent need to investigate the new pharmacological properties of myristicin-containing nutmeg oil. *Myristic fragrans* exhibit both antimicrobial and antifungal activity [28]. Nutmeg essential oils exhibit strong activity against parasites belonging to the phylum Apicomplexa (*Toxoplasma gondii*) *in vitro*, with low cytotoxic effects against normal cell lines [29]. *Myristic fragrans* methanolic extract reportedly induced a significant reduction in the number of *T. gondii* tissue cysts in the brain, a slight reduction in inflammation in the brain, and a distorted architecture of tissue cysts, as observed using scanning electron microscopy, in mice with toxoplasmosis [29]. Extraction is a crucial step in using medicinal plants. Solvent selection is mainly dependent on the specific properties of the targeted bioactive composites. Polar solvents are used to extract hydrophilic substances such as ethyl acetate, methanol, and ethanol [30].

This study aimed to investigate the efficacy of *M. fragrans* methanolic extract in controlling *C. parvum* infection in comparison with NZX (an FDA-approved drug control) in immunosuppressed and immunocompetent mice.

## Materials and Methods

### Ethical approval

The experiment was carried out in accordance with the guidelines set out by the International Animal Ethics Committee and Institutional Ethics Committee. The study protocol was approved by the Health Products Regulatory Authority and National Research Center Ethical Committee (No. 1473042022).

### Study period and location

The study was conducted during October and November 2023 (51 days) at the Animal House of the National Research Centre, Egypt.

### Experimental mice

One hundred laboratory-bred, 3–4 weeks old male Swiss albino mice were used in this study. Mice were maintained according to guidelines and standard protocols. The mice were acclimatized for 15 days before the experiment began. During this time, they were provided with a consistent supply of high-quality rodent food and clean water.

### Inoculation of *C. parvum* oocysts

We purchased *C. parvum* oocysts from the Theodore Bilharz Research Institute, Egypt. The oocysts were preserved in potassium dichromate (K_2_Cr_2_O_7_) until use for animal inoculation [31]. Just before induction of infection, the oocysts were washed at least 3 times through centrifugation in distilled water to separate the K_2_Cr_2_O_7_ until the solution became clear [32]. Eighty mice were orally infected with approximately 3 × 10^3^ *C. parvum* oocysts through an esophageal tube [33]. Fecal pellets collected from each group on day 8 p.i. were pooled, stained with modified Ziehl-Neelsen (MZN) stain (cold method), and examined to ensure successful infection of *C. parvum* oocysts [34, 35].

### Preparation of drug and plant extracts and dose adjustment

Dried seeds of *M. fragrans* were collected from a local market (Egypt) and identified by Prof. Enas H. Abdelrahman, Pharmacognosy Department, Faculty of Pharmacy, Cairo University, Kasr El-Aini Street, Cairo, Egypt. The methanolic extract of *M. fragrans* was prepared as described by Lin *et al*. [36]. The extract was liquefied in phosphate-buffered saline (PBS; pH 7.4) before oral administration to mice using an esophageal tube. The dose of *M. fragrans* extract was 500 mg/kg [37].

NZX was administered as nanazoxid tablets (500 mg) (Utopia Pharma, Egypt). The tablets were crushed, liquified in double distilled water (H_2_O), and administered orally using an esophageal tube. NZX was administered at a dose of 100 mg/kg (200 μL/mouse) [38]. All drugs were administered starting on day 9 p.i. for 5 consecutive days.

### Analysis of *M. fragrans* using gas chromatography-mass spectrometry

The nutmeg methanol extract was prepared by hydrodistilling nutmeg seeds using the conventional steam distillation method [39]. One microliter of *M. fragrans* essential oil was dissolved in 1 mL of methanol. Thereafter, 1 μL of the solution was added to the gas chromatography-mass spectrometry (GC-MS) mixture.

The GC/MS AgilentSeries (6890) (Thermo Fisher Scientific, Italy) was used with capillary columns (HP-5MS) (30 cm × 0.25 mm and 0.1 mm film thickness) of internal diameter 30 cm × 0.25 mm and 0.25 μm film thickness. The carrier gas (helium) pressure was 65 kPa. One microliter of the oil was introduced at a split ratio of 1:25 and a solvent delay of 2 min. The oven temperature was programmed as follows: Increasing from 60°C to 240°C at a rate of 3°C/min.

### Induction of immunosuppression

Fifty mice were orally administered dexamethasone through an esophageal tube. Each mouse was administered 0.25 mg/g/day dexamethasone dissolved in 200 mL of double distilled water for 2 weeks before the induction of *C. parvum* oocyst infection. Dexamethasone was continuously administered throughout the experimental period [40].

### Experimental groups

**Figure-1:**
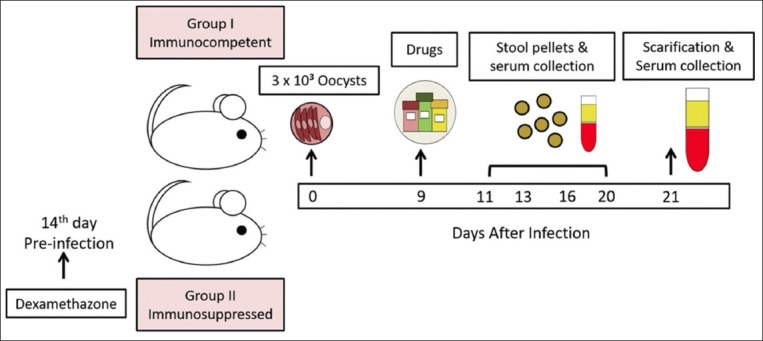
Infographic showing the treatment regimes, sampling timings, and sample processing.

Mice were divided into two groups (immunocompetent and immunosuppression) groups (Figure-1):

1. Group I (immunocompetent mice): 50 mice were further subdivided into the following groups:

- Group Ia (infection control): 10 infected and non-treated mice.
- Group Ib (drug control): 10 infected mice treated with NZX 100 mg/kg (200 μL/mouse) [38].
- Group Ic (study group): 10 infected mice treated with *M. fragrans* 500 mg/kg [37].
- Group Id (study group): 10 infected mice treated with a combination of NZX (100 mg/kg) and *M. fragrans* (500 mg/kg), maintaining the same total volume of 200 μL/mouse.
- Group Ie (negative control): 10 naïve mice (neither infected nor treated).

2. Group II (Immunosuppressed group): 50 immunosuppressed mice orally administered dexamethasone were further subdivided into the following groups:

- Group IIa (infection control): 10 infected and untreated immunosuppressed mice
- Group IIb (drug control): 10 infected immunosuppressed mice treated with NZX 100 mg/kg (200 μL/mouse) [38].
- Group IIc (study group): 10 infected immunosuppressed mice treated with *M. fragrans* 500 mg/kg [37].
- Group IId (study group): 10 infected immunosuppressed infected mice treated with a combination of NZX (100 mg/kg) and *M. fragrans* (500 mg/kg), maintaining the same total volume of 200 μL/mouse.
- Group IIe (negative control): 10 naïve mice (neither infected nor treated).

All mice were sacrificed on day 21 p.i. Mice were intraperitoneally administered thiopental (500 mg/kg) combined with heparin (100 units/mL) (anesthetic anticoagulant) through intraperitoneal injection [41].

### Parasitological evaluation

Parasitological was examined at 2-, 4-, 7-, and 11-days post-treatment (dpt). Stool samples were weighed and dissolved in 7% formalin. Thereafter, 50 μL of each sample was spread on a glass slide and stained with MZN using the cold method [35, 42]. The stained slides were observed using a microscope (Labomed, Model CXL, U.S.A.) with an oil immersion lens (oil 100×) to determine the mean number of *C. parvum* oocysts among different groups (mean number/10 microscopic fields). The mean number of oocysts/g of feces was calculated for each group [43]. The percent reduction (PR) in oocyst shedding was calculated as follows [3]:


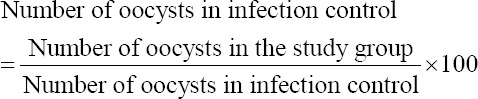


Percentage reduction in the shedded oocysts (PR) = Number of the oocysts in the infection control group – Number of oocysts in the study group/number of oocysts in infection control group × 100.

### Immunological evaluation

#### Antigen preparation

*C. parvum* oocysts were used for antigen preparation as described by Xiao *et al*. [44]. Fecal pellets were centrifuged using the sucrose and Percoll method for oocyst purification, followed by the addition of 0.5% Na hypochlorite for 10 min at 4°C. The solution was washed with sterile H_2_O 4 times, and then PBS was added (2 × 10^8^ oocysts/mL). Homogenization was performed using sterile PBS, followed by centrifugation for 30 min at 876 × *g*. Finally, the supernatant (containing the antigen) was stored (−20°C) until use. The Lowery method was used to measure protein content [45].

#### C. parvum -specific IgG

Blood samples were collected at 4 and 11 dpt and after sacrifice. An enzyme-linked immunosorbent assay (ELISA) was performed to determine the total specific IgG response in mice treated with *M. fragrans* methanolic extract. IgG was assessed as described by Oldham [46]. The checkerboard titration method was used to determine the optimum serum dilution and secondary conjugated antibody and antigen concentrations. The optical density cutoff points were determined according to Almazán *et al*. [47].

#### Cytokine measurement

The serum IFN-γ and interleukin (IL)-4 were measured at 21 dpt using a mouse ELISA kit (Biovision, Bejine, China). The assay was performed according to the manufacturer’s instructions. The samples were assayed in triplicate.

### Statistical analysis

The mean ± standard deviation of the numerical data was calculated. The statistical significance of differences among groups was assessed using one way analysis of variance and *post hoc* tests. Results with p < 0.05 were considered significant. GraphPad software (Version 6; GraphPad Software, Inc., La Jolla, CA, USA) was used for all statistical analyses.

## Results

### GC-MS analysis of the *M. fragrans* methanolic extract

The GC-MS analysis of the *M. fragrans* methanolic extract revealed 40 constituents, accounting for approximately 75% of the total *M. fragrans* weight. Myristicin (23.29%) was the major constituent, with methyl eugenol (2.6%), isoeugenol (3.3%), and other minor constituents. The compounds detected in the nutmeg oil are summarized in Table-1.

**Table-1:** Chemical composition of essential oil from nutmeg (*M. fragrans*) seeds.

Retention time (minutes)	Percentage	Identified compounds
10.74	0.18	1,3-cyclopentadiene, 1,2,3,4,5-pentamethyl
11.21	0.03	Sabinene
12.05	0.08	3,5-Dimethylanisole
12.89	0.02	Hydrocinnamaldehyde
15.90	0.05	5-caranol
16.38	0.05	Endobornyl acetate
19.26	0.23	(4R,5S)-1-Ethoxy-4-me thoxy-5-[(4-methoxyben zyl) oxy] hept-1-yn-6-ene
20	0.56	2-methoxy-4-(2-propen yl)
21.22	0.16	Geranyl acetate
22.13	**2.6**	Trans-methyl isoeugenol
22.28	0.09	1-Trimethylsilylhexa-1, 5-diyn-3-ol
23.63	**3.3**	Isoeugenol
24.85	0.07	Cadinene
24.95	0.3	Dodecanoic acid methyl ester
25.28	0.27	Croweacin
25.80	**23.29**	Myristicin
26.03	**4.5**	3-Hydroxy-1-(methylth io)-6,7,8,9-tetrahydro-5 H-benzocycloheptene
26.73	**9.4**	2-methyl-1,3-diphenyl2-(p-tolylsulfoxo) aziridine
26.79	0.16	-Spathulenol
27.08	0.13	Guaiol
27.41	**3.3**	3-Hydroxy-1-methylpropyl diphenylacetate
27.80	0.05	Isoaromadendrene epoxide
28.33	1.8	Methyl ester of 2,2,5,6-Tetramethylben zotetrahydrofuran-3-carboxylic acid
28.51	0.3	Eudesmol
29.04	0.02	α-bisabolol
29.83	**13.45**	Methyl tetradecanoate
30.31	0.02	Uvidin A
30.82	0.31	2-pentafluorophenylpro panal
31.01	0.07	Tetradecanoic acid, ethyl ester
31.80	0.12	Spiro[4.5]decan-6-ol, 6-methyl
32.31	0.04	1,3-Dimethyl-4,8-dioxa tricyclo[5.1.0.0 (3,5)]oc tane-2,6-diol
33.02	11.99	-Isopropyl-4-methyl-1,6,7,8-tetrahydro-2H-< as>-indacen-3-one
33.92	1.6	Hexadecenoic acid methyl esters
35.75	0.12	tetradecanamide
36.22	0.04	Tetradecanoic acid, 2-hydroxyethyl ester
37.29	3.9	5-Octadecadienoic acid, a methyl ester
38.32	0.06	-9-Octadecenoic acid ethyl ester
40.63	0.14	Methyl 9-eicosenoate
41.08	0.39	4-Acetoxy-2,3-dihydro5-hydroxy-2,2-dimethy l-1H-benz[g] indole
41.90	0.02	1-Heptatriacotanol
42.34	0.05	Kaurene
44.28	0.03	Heptatriacotanol
44.38	0.05	Benzene dicarboxylicacid
44.94	0.03	Fenchone
45.79	0.75	3-Methyl-2-nitro-N-pro pylaniline
47.22	0.1	1 (3H)-Isobenzofuranon e, 6,7-dimethoxy-
47.71	4.3	-5-Methyl-2,3,4,6-tetraphenyl-1,2,3,6 -tetrahydropyrimidine
48.80	2.8	-6-Methoxy-2-m ethyl-3-oxo-3,4-dihydro -2H-1,4-benzoxazine-2- carboxylic acid

*M. fragrans*=*Myristica fragrans*. The bold value represents the percentage of identified compound relative to the total identified compounds of the oil

### Parasitological evaluation

**Figure-2:**
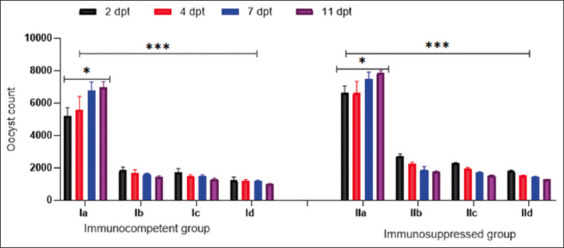
Count of *Cryptosporidium parvum* oocysts in different groups. Data are shown as mean ± standard deviation. *** and * Statistically significant (p < 0.001, and p < 0.05 respectively) using one-way analysis of variance. dpt=Days post treatment.

The number of oocysts shed in the group IIa was significantly higher (p < 0.001) than that of the group Ia throughout the experiment (Figure-2). The NZX-treated groups, the Ib and IIb groups, showed a significant gradual decline in the oocyst count starting from 4 and 11 dpt, achieving PR values of 79.7% and 77.7%, respectively. At 11 dpt, *M*. *fragrans* treatment reduced the oocyst count in the Ic group (PR = 81%. In addition, the IIc group showed a significant reduction (p < 0.001) in the oocyst count, with a PR of 80% versus 77% in the IIb group. The groups treated with a combination of NZX and *M. fragrans* showed the best results, with a PR of 85% in the Id group and 83% in the IId group (Table-2).

**Table-2:** Comparison of oocyst count and oocyst reduction percentage (%) between different groups in immunocompetent and immunosuppressed groups at different days post-treatment.

Days post-treatment	Immunocompetent group						

	Ia	Ib		Ic		Id	
			
	Mean × 10^2^ ± SD	Mean × 10^2^ ± SD	%Reduction	Mean × 10^2^ ± SD	%Reduction	Mean × 10^2^ ± SD	%Reduction
2	52.2 ± 5.2^bcd^	18.7 ± 1.9*	64.2	17.2 ± 2.4*	67.1	12.3 ± 1.9*	76.5
4	55.7 ± 8.2^bcd^	16.9 ± 1.9*	69.54	15.1 ± 0.7*	72.9	12.2 ± 0.7*	78.1
7	67.8 ± 5.4^bcd^	16.2 ± 0.6*	76.1	14.9 ± 0.8*	77.9	12 ± 0.5*	82.2
11	69.6 ± 3.6^bcd^	14.2 ± 0.9*	79.7	13.1 ± 0.5*	81.2	10.1 ± 0.3*	85.5

**Days post-treatment**	**Immunosuppressed group**						

	**IIa**	**IIb**		**IIc**		**IId**	
			
	**Mean × 10^2^ ± SD**	**Mean × 10^2^ ± SD**	**%Reduction**	**Mean × 10^2^ ± SD**	**%Reduction**	**Mean × 10^2^ ± SD**	**%Reduction**

2	66.2 ± 4.3*^BCD^	27.4 ± 1.3**^BCD^	58.6	22.7 ± 0.7**^D^	65.6	18.1 ± 0.6**^Db^	72.7
4	66.3 ± 7.3*^BCD^	22.4 ± 1.1**^CD^	66.1	19.8 ± 0.6**^D^	70.1	15.1 ± 0.4**^b^	77.2
7	74.9 ± 4.2*^BCD^	18.7 ± 2.1**^D^	75	17.3 ± 0.4**	76.9	14.4 ± 0.5**	80.7
11	78.6 ± 1.6*^BCD^	17.5 ± 0.7**^D^	77.7	15.3 ± 0.4**	80.5	12.8 ± 0.2**	83.7

Data are shown as mean ± standard deviation.

* Statistically significant (p < 0.001) in comparison with group Ia,

** Statistically significant (p < 0.001) in comparison with group IIa, ^B^Statistically significant (p < 0.001) in comparison with group Ib, ^C^Statistically significant (p < 0.001) in comparison with group Ic,

D Statistically significant (p < 0.001) in comparison with group Id,

b Statistically significant (p < 0.001) in comparison with group IIb, ^c^Statistically significant (p < 0.001) in comparison with group IIc, ^d^Statistically significant (p < 0.001) in comparison with group IId

### *C. parvum*-specific IgG examination

**Figure-3:**
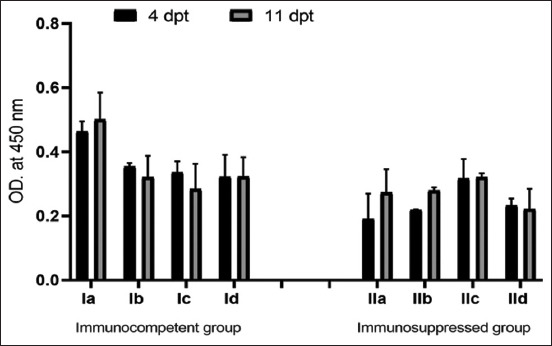
Optical density of serum immunoglobulin G against *Cryptosporidium parvum* oocyst antigen in the experimental groups. Data are presented as mean ± standard deviation. dpt=Days post treatment

The IgG level in the dexamethasone-treated IIa group was lower (p < 0.05) than that in the Ia group at 4 and 11 dpt. In the Ib group, IgG levels were significantly higher at 4 dpt than at 11 dpt; however, IgG levels at both 4 and 11 dpt were significantly lower (p < 0.05) than those in the Ia group. *M. fragrans* methanolic extract successfully decreased IgG levels in groups Ic and IIc at 11 dpt (Figure-3). However, the combination of the drug and *M. fragrans* methanolic extract decreased IgG levels at both 4 and 11 dpt, and IgG levels were significantly lower in the IId group than in the Id group.

### Cytokine measurements

**Figure-4:**
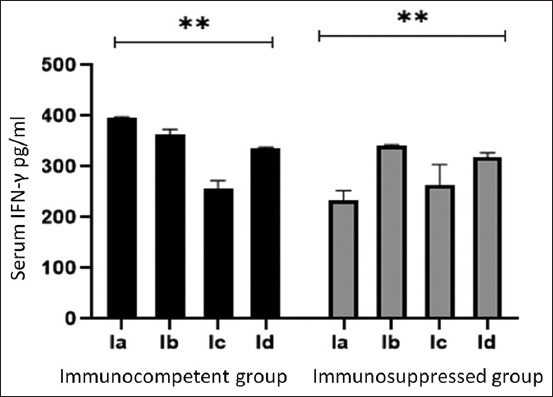
Interferon-gamma serum level in different experimental groups of immunocompetent and immunosuppressed mice. Data are expressed as mean ± standard deviation. **Statistically significant (p < 0.001) using one-way analysis of variance.

**Figure-5:**
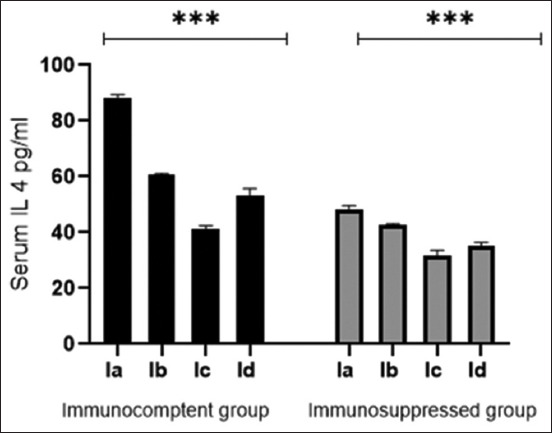
Interleukin 4 serum level in different experimental groups of immunocompetent and immunosuppressed mice. Data are expressed as mean ± standard deviation. *** Statistically significant (p < 0.001) using one-way analysis of variance.

The IFN-γ and IL-4 levels were lower in the IIa group than in the Ia group. *M. fragrans* levels were significantly decreased in the Ic, Id, IIc, and IId groups (p < 0.001) on day 21 p.i. compared with the corresponding controls (Figures-4 and 5).

## Discussion

The effects of *M. fragrans* may be due to the active ingredients in the methanolic extract. Gas chromatography–mass spectrometry analysis of *M. fragrans* essential oil in this study revealed 40 bioactive compounds. Myristicin was the major constituent (23.29%). Myristicin may be responsible for the anti-inflammatory properties of *M. fragrans*. It inhibits the secretion of nitric oxide, chemokine, cytokines, and growth stimulators in dsRNA-enhanced macrophages through the Ca^+2^ pathway [48]. In this study, isoeugenol and methyl eugenol accounted for 3.3% and 2.6% of all components, respectively. Eugenol inhibits cellular glucose uptake, affects membrane permeability, and reduces energy production [49]. *M. fragrans* methanolic extract has been found to possess therapeutic efficacy without safety issues [50–52].

In our study, *C. parvum* oocyst shedding started on day 8 p.i. and continued in large numbers in Group IIa mice until day 21. This is in agreement with the findings of Sonzogni-Desautels *et al*. [53], who found that oocyst excretion began around days 5 and 7 p.i. and persisted until day 30 p.i. Mahmood *et al*. [54] reported that the highest number of oocysts was shed in the feces of infected immunocompromised mice at 20 dpt. Benamrouz *et al*. [33] also showed that the shedding of oocysts reached >10,000 oocysts/g of feces in mice inoculated with low-dose oocysts on day 45 p.i.

In the present study, the parasitological evaluation results revealed a significant decrease (p < 0.001) in *C*. *parvum* oocyst shedding in the infected groups treated with *M. fragrans* alone or in combination with NZX (Ic, IIc, Id, and IId groups). The PR for oocyst shedding in stools was greater in groups Ic, IIc, Id, and IId than in groups Ib and IIb, which received NZX alone. In addition, the levels of IgG and both studied cytokines decreased on day 21 p.i., approaching those in the corresponding uninfected groups. In contrast, NZX treatment failed to reduce IgG and cytokine levels. Our results agree with those of Pillai *et al*. [55], who tested *M. fragrans* extract against *T. gondii* tachyzoite and reported significant inhibition of *T. gondii* tachyzoite development (p < 0.01) in a dose-dependent manner. Moreover, *M. fragrans* was found to have lethal effects on the Anisakis roundworm in the infective stage [56].

The present study demonstrated the superiority of a combination of NZX and *M. fragrans* for treating cryptosporidiosis over the use of either drug alone, although no significant differences were observed among the groups. Using drug combinations helps to synergize the efficacy of drugs, thereby reducing the required dose and decreasing the occurrence of drug resistance [57]. Similarly, Esmat *et al*. [58] reported that NZX monotherapy had the lowest anticryptosporidial efficacy, whereas the combination of clofazimine and NZX resulted in significant parasite clearance in immunosuppressed mice. Moreover, NZX has been proven to be inefficient in patients with acquired immunodeficiency syndrome, organ recipients, and undernourished children; thus, it needs to be combined with other anti-parasitic drugs, such as azithromycin and rifaximin [59].

*M. fragrans* exhibits antimicrobial activity against *Helicobacter pylori*, *Bacillus subtilis*, multidrug-resistant *Salmonella typhi*, *Escherichia coli*, and *Saccharomyces cerevisiae* [13]. Nutmeg oil affects the growth and survival of *E. coli*, *Listeria monocytogenes*, *Yersinia enterocolitica*, and *Staphylococcus aureus* [51]. The antifungal activity of *M. fragrans* against *Aspergillus glaucus*, *Aspergillus niger, Colletotrichum gloeosporoides*, *Colletotrichum musae*, *Fusarium semitectum, Fusarium oxysporum*, and *Candida albicans* has been demonstrated [60, 61]. The ethanolic extract of *M. fragrans* was investigated against *T. gondii* *in vivo* and found to have low efficacy [62].

Individuals with lower immunity, such as those receiving cancer chemotherapy, those infected with HIV, children, and those on corticosteroids, are more susceptible to *C. parvum* infection with more severe disease manifestations [3]. Therefore, in this study, we assessed the efficacy of *M. fragrans* in treating cryptosporidiosis in immunosuppressed and immunocompetent mice. Dexamethasone was used for immunosuppression induction at a dose selected according to Rehg *et al*. [40], who tested several doses of dexamethasone and recommended 0.25 μg/g/day for 14 days preceding infection. They reported that higher dexamethasone doses could result in unacceptable toxicity, whereas lower doses were not effective.

Dexamethasone inhibits Th1 cytokine secretion more than Th2 cytokine secretion [63]. Th1 lymphocytes are the main cells secreting IFN-γ, whereas Th2 lymphocytes secrete IL-4 [39]. IFN-dependent mechanisms limit cryptosporidiosis infection [64]. Moreover, IL4 plays a key role in *C. parvum* infection because it can activate the production of antibodies through the activation of immunological memory B cells [65]. IL-4 and IFN-γ have a synergistic effect in alleviating cryptosporidiosis [66]. Moreover, IL-4 expression is not correlated with oocyst secretion or disease symptoms [67]. In this study, this finding was evident in the IIe group with significantly lower IFN-γ level than that in the Ie group. However, no significant difference in IL-4 levels was observed between the groups. In contrast, cryptosporidiosis caused significant elevations in the levels of the assessed cytokines (IFN-γ and IL-4). Moreover, their levels significantly decreased at 18 dpt when *M. fragrans* extract. An increase in the IFN-γ level was expected in the early stage of infection to facilitate parasite clearance. However, in the late stage (21 days p.i.), on treatment with M. fragrans, which has anti-inflammatory properties, the level of IFN-γ decreased. The present study result complied with that of Poorbagher *et al*. [68], who indicated that *M. fragrans* has an anti-inflammatory effect, as evidenced by decrease in the levels of IFN-γ and IL-4. In addition, Farid *et al*. [38] recorded high levels of cytokines (IFN-γ, IL-4, IL-6, IL-10, and IL-17) on day 7 p.i. and continued to reach the highest level on day 14 p.i.; use of *Aloe vera* as treatment successfully decreased all cytokine levels on days 21 and 32 p.i.

## Conclusion

Oral administration of *M. fragrans* reduced *C. parvum* oocyst shedding by 81% and 80% in immunocompetent and immunosuppressed mice, respectively. In addition, it exhibited anti-inflammatory properties, reducing serum levels of IgG, IFN-γ, and IL-4. Additional research should be performed to determine the active ingredients of *M. fragrans*, their concentrations, and extraction.

## Authors’ Contributions

EHAR, RSZ, and EEE: Designed the study. EHAR: Monitored the design and coordination of the study. EHA: Prepared plant extracts and performed analysis of *M. fragrans* (GC-MS). EEE, FA, NMT, and RSZ: Designed and conducted the study. EEE and EHAR: Measured the immunological parameters. EEE, NMT, and FA: Data analyses and interpretation. NMT and EEE, FA: Wrote the original manuscript. All authors have read and approved the final manuscript.
